# How Do Trees Grow in Girth? Controversy on the Role of Cellular Events in the Vascular Cambium

**DOI:** 10.1007/s10441-021-09418-y

**Published:** 2021-06-21

**Authors:** Anna Wilczek-Ponce, Wiesław Włoch, Muhammad Iqbal

**Affiliations:** 1 Department of Biosystematics, University of Opole, Oleska 22, 40-052 Opole, Poland; 2 Polish Academy of Sciences Botanical Garden – Centre for Biological Diversity Conservation in Powsin, Polish Academy of Sciences, Prawdziwka 2, 02-973 Warsaw, Poland; 3 Department of Botany, Hamdard University, Tughlaqabad, New Delhi, 110 062 India

**Keywords:** Cambial circumference, Elimination of initials, Initial surface, Intrusive growth, Symplastic growth

## Abstract

Radial growth has long been a subject of interest in tree biology research. Recent studies have brought a significant change in the understanding of some basic processes characteristic to the vascular cambium, a meristem that produces secondary vascular tissues (phloem and xylem) in woody plants. A new hypothesis regarding the mechanism of intrusive growth of the cambial initials, which has been ratified by studies of the arrangement of cambial cells, negates the influence of this apical cell growth on the expansion of the cambial circumference. Instead, it suggests that the tip of the elongating cambial initial intrudes between the tangential (periclinal) walls, rather than the radial (anticlinal) walls, of the initial(s) and its(their) derivative(s) lying ahead of the elongating cell tip. The new concept also explains the hitherto obscure mechanism of the cell event called ‘elimination of initials’. This article evaluates these new concepts of the cambial cell dynamics and offers a new interpretation for some curious events occurring in the cambial meristem in relation to the radial growth in woody plants.

## Introduction

**Fig. 1:**
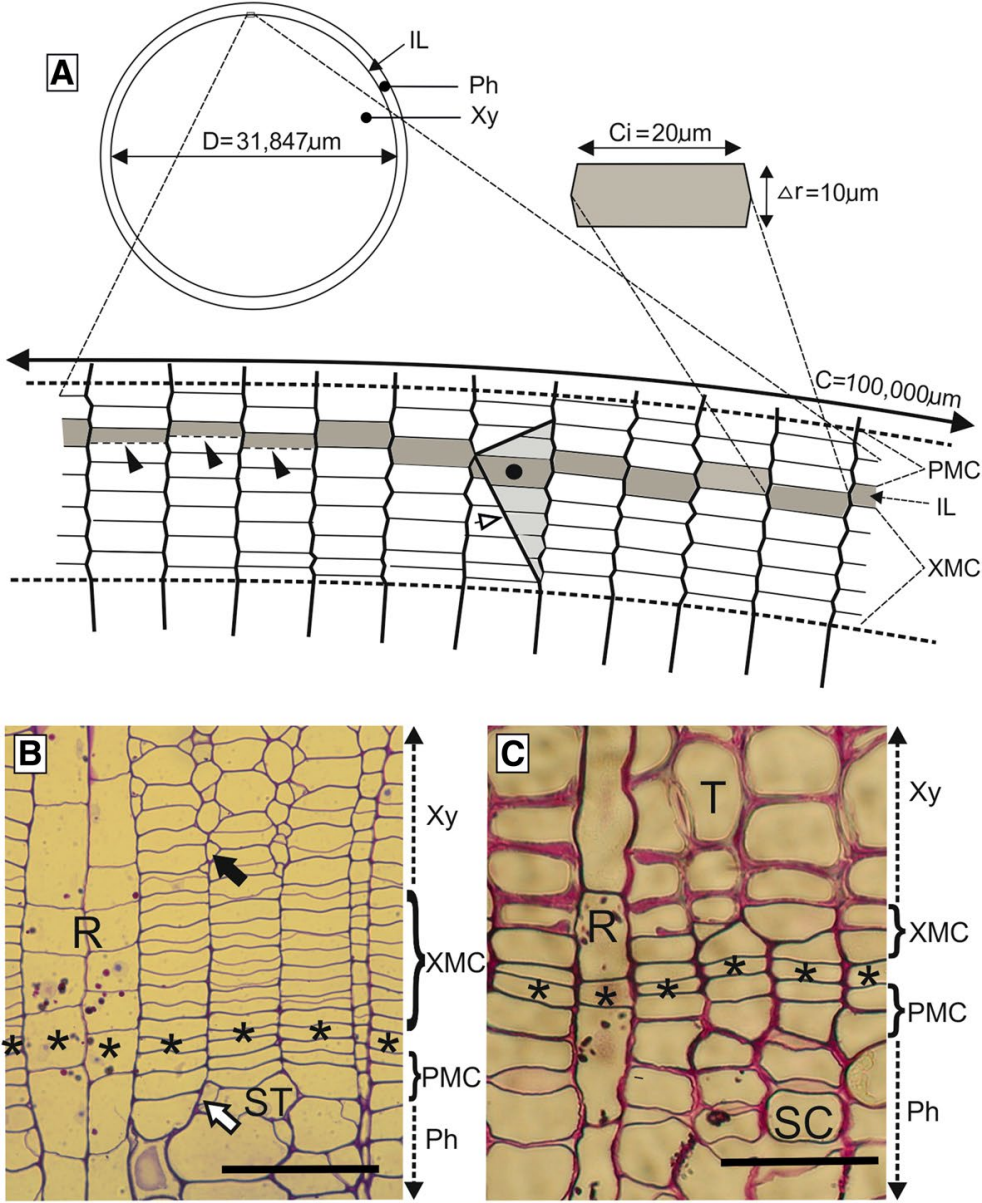
Vascular cambium in a transverse section of stem. **A** Schematic of a cross-section of stem axis, showing correlation between diameter and circumference of cambial cylinder. Slanted walls, indicating transformation of periclinal walls into radial ones, pointed with a white arrowhead, are typical for the area of intrusive growth and elimination of initial. The little rectangle marks the position of the enlarged fragment underneath. One initial has been enlarged to exhibit its typical dimensions. Dashed lines indicate the cambium zone; initial cells are marked with grey. The initial marked with a black circle has increased its circumferential dimension due to intrusive growth, but the change has been compensated with an equal partial elimination of the neighbouring initial. IL—initial layer, Ph—phloem, Xy—xylem, PMC—phloem mother cells, XMC—xylem mother cells. As the diameter of cambial cylinder (D) is 31,847 µm, its radius r = D/2 = 15,923.5 µm and circumference C = 2πr = 100,000 µm. Similarly, if the average circumferential dimension (Ci) of a single cambial initial cell is 20 µm, the total number of cambial initials forming the circumference N = C/Ci = 100,000 µm/20 µm = 5000. Also, if the average radial dimension of an initial is 10 µm, increase in radius of cambial cylinder due to deposition of one cell layer on xylem side Δr = 10 µm, an increase in cambial circumference after addition of one cell layer to the xylem core is ΔC = 2πΔr = 6.28 × 10 µm = 62.8 µm. Moreover, increase in circumferential direction of each cambial initial ΔC_i_ = ΔC/N = 62.8/5000 = 0.0126 µm, and the ratio of radial growth to circumferential growth of wood (Δr/ΔC_i_) = 793.65. **B** Cross-section of *Tilia cordata.* PMC—phloem mother cells; XMC—xylem mother cells. Ph—phloem, Xy—xylem, R—ray, ST—sieve tube element. Black arrow indicates an intrusively growing wood fibre, whereas white arrow points to companion cell. Asterisks indicate the location of the most probable initial cells. **C** Cross-section of *Pinus sylvestris.* T—tracheid. SC—sieve cell. PMC—phloem mother cells; XMC—xylem mother cells; Ph—phloem, Xy—xylem, R—ray*.* Asterisks indicate the location of the most probable initial cells. Scale bars—50 μm

With an estimated global forest growing stock of 530.5 billion m^3^ (Köhl et al. 2015), production of wood and bark by the activity of vascular cambium, the lateral meristem in woody plants, is one of the most important biological processes on Earth. The cambium exists in a form of cylinder of multi-layered meristematic cells between xylem and phloem tissues (Fig. 1a–c). It surrounds the central wood core and is itself surrounded by an outer cylinder of bark in the long axis (root and shoot) of woody plants. In transverse sections of the plant axis, it appears as a multi-layered circle around the xylem, comprising of a division zone in the middle, where cell divisions occur, and the differentiation zones of peripheral layers, where derivative cells pass through a variety of processes on way to attaining their final form and position in derivative tissues. The division zone generally consists of a layer of cambial initials sandwiched by the layers of xylem mother cells (XMCs) on the inner side and those of phloem mother cells (PMCs) on the outer side (Fig. 1a–c).

Forests act as the major terrestrial carbon sinks, and possess large carbon pools mainly in the form of wood produced by the cambium activity in branches, stems and roots of trees (Mahli et al. 2011). Climate, or local atmospheric condition, is the main regulator of cambium activity and the consequent carbon allocation in woody parts of trees (Iqbal et al. 2010a, b; He et al. 2018). On being triggered by environmental factors (such as temperature level, water availability, air quality, light intensity and day length), some long-distance hormonal signals and short-range peptide signals jointly regulate the cambial activity. Communications from endodermis and phloem tissues also influence the proliferation of cambial initial cells (Iqbal et al. 2005; Krepkowski et al. 2011; Bosio et al. 2016; Wang 2020). Interactions between these signaling pathways render the vascular development flexible.

Xylogenesis, i.e. the process of production of new cells from cambium and their differentiation into mature functional wood cells, comprises of periclinal divisions of XMCs creating new daughter cells, enlargement of all these cells, deposition of cellulose and hemi-cellulose to form secondary cell walls, impregnation of cell walls with lignin, and finally the programmed cell death (Rathgeber et al. 2016). As new layers of wood cells are produced each year on the inner side of the cambial cylinder, increasing the diameter of the wood core, the circumference of the cambial cylinder is bound to increase. Surprisingly, many processes related to growth in radial direction (the wood-core thickness) and expansion of cambial circumference remain poorly understood.

Morphogenesis in plants is typically coordinated by organizer cells that direct the adjacent stem cells to undergo programmed cell division and differentiation. Using the lineage-tracing and molecular genetic studies in roots of *Arabidopsis thaliana*, Smetana et al. (2019) showed that the concept of organizer cells applies to cambium also, where cells with a xylem identity act as organizer cells and direct the adjacent cambial cells to divide and function as stem cells. Placing a genetic label on individual cambial cells and tracing their derivatives in the poplar stem, Bossinger and Spokevicius (2018) found that differentiation of xylem and phloem was not well synchronized and hence likely to be controlled independently. They observed a frequent loss of cambial initials but such a cell loss was rare in XMCs or PMCs. Further, the period for which the mother cells remained active, varied greatly, showing that the time or the number of cell cycles for which the mother cells remained active was not pre-determined. Through pulse labeling and genetically-encoded lineage tracing in active cambium of *Arabidopsis thaliana* hypocotyl, Shi et al. (2019) mapped the activity of cambial initials (identified by them as stem cells) and confirmed that a single bifacial cambial initial generates both xylem and phloem cell lineages. They established different transgenic markers, which defined a proximal, a distal and a central cambium domain, representing the site of xylem formation, the site of phloem formation and the site of strongly proliferating bifacial cambial initials (stem cells), respectively.

Studies undertaken on the cambial cell growth during the last two decades (Karczewska et al. 2009; Kojs 2012; Kojs et al. 2004a, b; Jura et al. 2006; Włoch et al. 2009, 2013; Wilczek et al. 2011a, 2018; Miodek et al. 2021) have led to a new hypothesis on the mechanism of intrusive growth of the cambial initials. These studies have elucidated certain characteristic features of the cambium from new angles, suggesting that the mechanical strains in the cambial tissue likely affect the processes involved in the formation of wood and the consequent increase in the cambial circumference. This article attempts to compare these newly emerging concepts with the traditional knowledge of the cambial dynamics, with special focus on radial growth of wood in stems and roots and the consequent expansion of the cambial circumference.

## The Structure of Vascular Cambium

Whereas only a few cells normally act as initials in apical meristems, cambial initials are extremely numerous and form a sort of layer between the layers of tissue mother cells (PMCs and XMCs) around the wood core of the trunk, branches and roots of woody plants (Fig. 1). They are responsible for the production of wood cells on the inner side (Ajmal and Iqbal 1987; Aref et al. 2014; Włoch et al. 2013) and secondary phloem cells on the outer side (Iqbal and Ghouse 1987; Iqbal and Zahur 1995). The layer of initials is not in fact a perfect layer due to the non-parallelism of its component cells (Włoch 1981), but is a significant demarcation plane between xylem and phloem from developmental point of view. This has also been described as ‘the initial surface’ (Włoch and Połap 1994; Włoch et al. 2013).

**Fig. 2:**
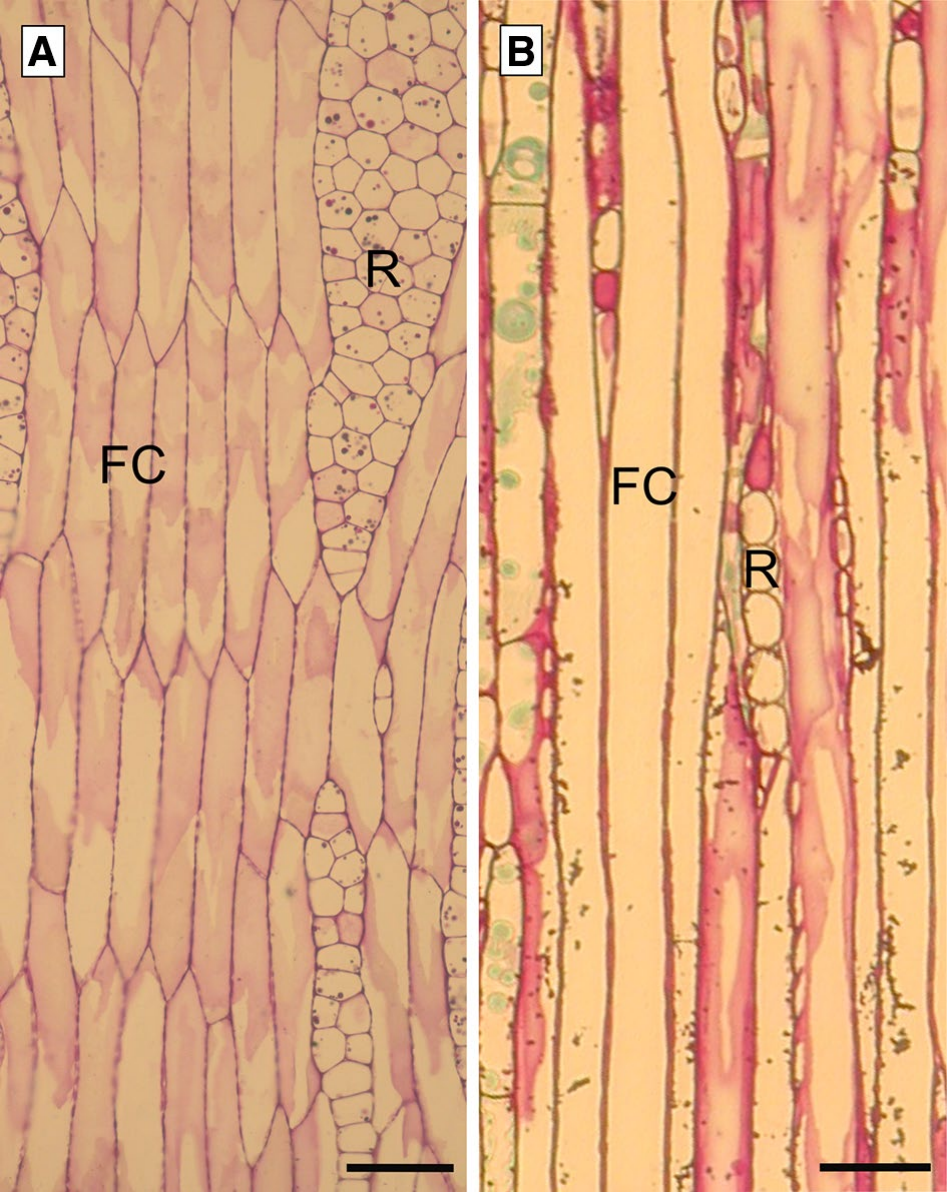
Tangential sections of storeyed (*Tilia cordata*) and nonstoreyed (*Pinus sylvestris*) cambium. FC—fusiform cells; R—ray. Scale bars—50 μm

Unlike the initial cells of primary meristems, which are normally homogeneous in shape, cambial initials are usually of two types: (a) axially elongate and highly vacuolated fusiform initials, and (b) relatively small, almost isodiametric or radially extended ray initials that often occur in aggregates (Fig. 2) (Iqbal and Ghouse 1990; Larson 1994; Evert 2006). Fusiform initials produce the axially aligned derivatives such as vessel elements, tracheids, fibres and sieve-tube elements of the secondary vascular tissues; whereas ray initials give rise normally to the radially aligned ray parenchyma. In transverse view, ray parenchyma appear to form radially running rays across the secondary vascular tissues, i.e. xylem and phloem (Fig. 1b, c) (Larson 1994; Iqbal 1995; Lev-Yadun and Aloni 1995). Cambial initials (stem cells) never undergo differentiation but continue to remain initials and produce tissue mother cells (Iqbal 1994, 1995; Lachaud et al. 1999).

Based on the form and arrangement of the initial cells, as seen in tangential longitudinal view, the cambium is identified to be storeyed or non-storeyed. It is non-storeyed, if the fusiform initials, relatively long and diversified in shape and length, terminate at varied height levels and neither the fusiform initials nor the rays are arranged in tiers, forming storeys (Fig. 2b) (Ghouse and Yunus 1974; Włoch and Szendera 1989; Evert 2006). On the contrary, the cambium is storeyed if the fusiform initials are relatively short in axial direction and terminate nearly at the same height, thus forming horizontal tiers placed one above the other (Fig. 2a); it is double-storeyed if both fusiform initials and rays (aggregates of ray initials) are arranged in storeyed fashion, forming horizontal bands. The storeyed structure develops ontogenetically from the non-storeyed one (Soh 1990; Larson 1994).

Cambial initials also undergo directional and dimensional changes unrelated to the formation of storeyed pattern, a feature termed as a ‘rearrangement of cambial initials’. This is considered as the mechanism for the formation of grain in the wood, and possibly affects the development of the vessel network (Zimmermann 1983). It seems that the rearrangement of cambial initials helps the cambial adaptation to diverse conditions of both the external and internal environments (Kojs et al. 2002). It is assumed that the processes contributing to cell rearrangement include anticlinal divisions, unequal or imperfect periclinal divisions, intrusive growth, elimination of initials, and changes in the ray pattern (Larson 1994; Evert 2006).

## Mechanical Strains in the Vascular Cambium

The cambial cells are exposed to mechanical stresses resulting not only from their turgor but also from the radial growth of secondary vascular tissues (Hejnowicz 1980, 1997; Kwiatkowska and Nakielski 2011). Radial growth of the wood core pushes the cambial tissue from the inside outwards, stretching the layer of cambial initials and creating conditions for intrusive growth of initials. This, together with the constraints imposed by the bark, generates compressive stress in radial direction, which is received by the cambial layers sandwiched between the wood and the bark (Iqbal and Ghouse 1990; Kwiatkowska and Nakielski 2011). The question of why a delicate and fragile tissue like cambium is not crushed under these circumstances, while the mechanical stress resulting from an increase in the wood’s radius is strong enough to cause an expansion in the bark circumference remains unanswered.

It is assumed that under specific conditions a tensile stress (in the radial direction) may occur in the vascular cambium, for instance in areas of phloem collapse, often during early spring. This phenomenon has been correlated to the enlargement of vessel-element mother cells (Hejnowicz 1997; Kwiatkowska and Nakielski 2011). However, in view of the massive growth of vessel members occurring in spring, it may be argued that it should not be based on unusual conditions. Further, no attention has been paid to numerous cases where intrusive growth of the initials is located close to the growing vessel members, sometimes separated by only a few layers of xylem derivatives. These two phenomena were explained by assuming the presence of two different mechanical conditions in the tissue, i.e. a radial compressive strain in the case of the intrusive growth of cambial initials along the radial walls, and a radial tensile strain in the case of the intrusive growth along the tangential walls of the vessel-element mother cells.

Studies in developmental plant biology and biophysics, together with a detailed analysis of a large number of anatomical sections of active cambium, have suggested a reassessment of the mechanical stresses existing in vascular cambium. Studies have also pointed out that the diurnal variation of water balance in plants, negative during the day and positive during the night, is of crucial importance in the process of radial increment of tissues (Kojs and Rusin 2011; Kojs 2012).

Transpiration is intense during the daytime, causing a strong negative pressure in vessels. Water flows into vessels from the surrounding living tissues, and hence the water potential of cells in this part of the plant goes down (Klepper 1968). The turgor pressure of tissues decreases and the whole organ (trunk, branch or root) shrinks (Ueda and Shibata 2001). These changes in turgor pressure occur in both the xylem (wood) and phloem (inner bark) (Alméras 2008). It is also likely that some preventive measures are induced in the living cells of the vascular tissues to protect these cells from excessive dehydration (Kojs and Rusin 2011). After the sunset, transpiration becomes less intense and water flows back into cells, increasing their water potential, and hence the turgor pressure (Klepper 1968).

As most of the wood cells are dead cells, with strong and often lignified cell walls, changes in the wood-core diameter due to variations in tissue hydration are less distinct than in the phloem, which consists dominantly of living and osmotically active cells (Molz and Klepper 1973). While measuring the different strains in the secondary xylem and phloem, Alméras (2008) and Alméras et al. (2006) noted that phloem layers exert pressure on the xylem cylinder, generating a compressive stress in the delicate meristematic tissue (vascular cambium) located between them, when the turgor pressure of phloem cells in the bark decreases (during the daytime). When the phloem cells regain their turgor (during the night), the phloem layers move away from the wood cylinder, thus generating a tensile stress in the radial direction and causing a radial stretching of cambial cells between the phloem and xylem. The major part of this alteration in shape and size of the cambial cells (elastic deformation) reverses with the beginning of a new day, but a small part of it is retained (plastic deformation), which may be regarded as the net radial increment in the cambial cell dimension (Kojs and Rusin 2011).

## Divisions of Cambial Cells

### Periclinal Divisions

Cambial cells frequently undergo periclinal cell divisions that occur parallel to the closest organ surface and result in the addition of derivative cell layers. Occasionally, they also experience anticlinal divisions that occur perpendicular to the closest organ surface, adding new cells to the layer of initials and forming new radial files (Barlow et al. 2002). Occurrence of anticlinal divisions normally remains confined to the layer of initials, but periclinal divisions occur both in the initial layer as well as in layers of XMCs and PMCs (Butterfield 1975).

The main activity of cambial cells is their expansion in the radial direction followed by their periclinal division, which reduces the radial thickness of the cell to almost half, but the tangential width remains unaffected (Fig. 1a). The resultant daughter cells, having a radial thickness about half of the thickness of the mother cell, expand radially to regain the original thickness of the mother cell before undergoing the next periclinal division, thus forming a radial file of cells (Bailey 1923; Philipson et al. 1971). After the periclinal division of an initial cell, one of the daughter cells maintains the ‘initial’ status, while the other one acts as a xylem or phloem mother cell, depending on whether it is located inside or outside the initial surface, respectively. While the initial cells maintain their meristematic nature, the xylem or phloem mother cells usually leave the cambial zone after several periclinal divisions and begin to differentiate into xylem elements (tracheids, vessel elements, parenchyma and fibres) or phloem elements (sieve-tube elements, albuminous or companion cells, parenchyma and fibres) (Iqbal 1994, 1995; Larson 1994; Evert 2006).

Periclinal divisions, together with symplastic growth of cell walls in the radial direction, are considered to be the cause of radial growth in woody plants (Evert 2006). An increase in cell number due to the occurrence of periclinal divisions as such has no direct influence on the radial dimensions of cambial zone, because the increase in cell number is accompanied by a decrease in the radial dimension of the cells. It is the symplastic growth taking place between two successive periclinal divisions, which actually increases the radial dimension (thickness) of the tissue (cambial zone).

As fusiform cambial initials are axially elongated and radially flattened cells (see Figs. 1, 2), such cells should divide by transverse division as per the Errera’s rule (Kwiatkowska and Nakielski 2011). However, cambial cells divide predominantly by periclinal divisions. Transverse divisions with a minimal cell-plate surface are rare in vascular cambium and occur mainly during ray formation (Cumbie 1967; Evert 2006).

Mechanical strains play an important role in control of cells division and differentiation of plant cells, and nuclei are sensitive to the frequent external mechanical stimulation (Qu and Sun 2008). As per the unified hypothesis of mechano-perception in plant cells proposed by Telewski (2006), the role of mechanical stimuli in plant morphogenesis is certain and beyond any doubt. Studies of isolated plant protoplasts have revealed that the cell-plate orientation depends on the pattern of mechanical strains; it is usually parallel to the orientation of the principal compressive tensors, although in some cases it is perpendicular (Lintilhac and Vesecky 1984; Lynch and Lintilhac 1997). As mentioned earlier, it is commonly accepted that cambial cells are radially compressed (Kwiatkowska and Nakielski 2011), which means that the cell plate should be formed predominantly parallel to the radius, and hence frequent anticlinal divisions should be expected. However, it is the periclinal division that occurs most frequently in the cambial cells (Lintilhac and Vesecky 1984; Iqbal 1994; Lynch and Lintilhac 1997).

Considering the probable relation between the compressive stress and the cell-plate orientation (parallel to each other), one might think that if periclinal divisions are dominant, the predominant compressive tensor should be oriented in the tangential plane of cambial cells. However, this idea counters most of the reports hitherto made and hence is not viable. Studies on protoplasts suggest that compression in one direction causes tension in the plane perpendicular to the axis of the compression (Lintilhac and Vesecky 1984; Lynch and Lintilhac 1997). A study by Louveaux et al. (2016) has revealed that the tensile stress defines the orientation of the division plane, i.e. division plates are located along the local maximum tensile stress in cell walls. The tensile stress in the radial direction, which stretches the radial walls of cambial cells, may likely create a local maxima of tensile stress in these walls, determining the periclinal division of cambial cells. However, such a possibility has hardly been examined so far. Future research in this direction should explain the cause of the peculiar orientation of periclinal divisions of cambial cells, which might stem from the tensile stress that occurs in the radial direction.

Another hypothesis suggests that the division plane in cells of apical meristems is normal to the main growth direction (Hofmeister 1863). This rule may also be applied to cambial cells, because their maximal growth occurs in the radial direction. The modification of Hofmeister’s rule takes into consideration the principal growth tensor (Hejnowicz and Romberger 1984). However, we have no argument to explain why both the maximal growth direction and the maximal tensor occur in a direction in which the cambial tissue is compressed.

Considering the new reports on cambial cell dynamics (Kojs and Rusin 2011; Kojs 2012; Włoch et al. 2013) it seems plausible that the radial direction of maximal growth, as well as the maximal growth tensor, may be an outcome of tensile stress in the radial direction that occurs in the cambial tissue during the night time. In this context, studies on the cambial tissue culture in vitro should not be lost sight of. The cambial cells grown in vitro do not exhibit radial expansion. They assume isodiametric shape, which possibly suggests that the shape of fusiform cells is also an outcome of the specific mechanical environment inside the plant (Brown 1964; Brown and Sax 1962).

Based on the original work of Mahmood (1968), it has been described by many subsequent workers, including Murmanis (1970, 1977), that after each periclinal division a new primary cell wall develops around the two daughter protoplasts. As a result of the ‘emboxing’ phenomenon described by Mahmood (1968), the inner tangential walls of the periclinally dividing initials, which grow considerably thick during phloem formation, become part of the first derivatives on the xylem side when xylogenesis starts. Similarly, the outer tangential walls that thicken gradually during xylem formation, become part of the first derivatives on the phloem side when leptogenesis begins again. This is how the thickness of the tangential walls of cambial initials is maintained during the deposition of secondary vascular tissues. Murmanis (1970), using electron microscopy, confirmed the difference in the thickness of the inner and outer tangential walls of the dividing initials during the phloem and xylem formation. Catesson and Roland (1981), however, observed the deposition of new primary wall only in the area of the developing tangential cell plate. This possibly suggests that the thickening of walls may occur during cytokinesis, interphase or the whole cell cycle.

On the other hand, the radial walls keep receiving the extra depositions continuously, with each division of the initial, irrespective of whether tissue formation occurs on the outer or the inner side of the cambial initials. However, the thickness of these walls is simultaneously reduced by their successive stretching in the radial direction during the symplastic expansion of cells after each periclinal division. Thus, thickness of radial walls remains more or less uniform due to addition of wall material on one hand, and radial extension of the wall on the other. In general, radial walls are significantly thicker than tangential ones. Assuming that cambial cells are tensed in the radial direction during the night, which for the most part is an elastic deformation (Kojs and Rusin 2011; Kojs 2012), the thickness of radial cell walls may perhaps be an adaptation to this plausibly strong tension.

### Anticlinal Divisions of Cambial Cells

Any increase in the girth of the wood cylinder (i.e. radial growth) necessitates a corresponding increase in the cambial circumference, which is made possible through anticlinal divisions of cambial initials and a meager symplastic growth in the circumferential direction (Karczewska et al. 2009; Włoch et al. 2013; Miodek et al. 2021). The expansion of cambial initials in the circumferential direction, and their anticlinal divisions, help maintain the more or less constant cell dimensions, except during the first few years of the cambial activity when fusiform initials increase their tangential dimensions, especially in both storeyed and non-storeyed cambium (Srivastava 1973; Larson 1994). During formation of storeyed structure tangential dimensions, especially their length, slightly decreases together with unification of their length (Kojs et al. 2004b; Wilczek 2012).

When cambium cylinder increases its circumference, occurrence of longitudinal anticlinal divisions is just expected. Several types of anticlinal division (oblique, longitudinal, lateral) have been described in the relevant literature (Iqbal and Ghouse 1990; Larson 1994). Oblique anticlinal (pseudotransverse) divisions are predominant in non-storeyed cambia, whereas longitudinal anticlinal divisions characterize the storeyed cambium (Cumbie 1963, 1967, 1984; Butterfield 1972; Krawczyszyn 1977; Rao and Dave 1985). In the mosaic type of cambium, typical for transition from a non-storeyed structure to the storeyed one, anticlinal division is of an intermediate type, wherein the length of the wall normally covers more than 50% but less than 70% of the total cell length (Krawczyszyn 1977). Lateral divisions of fusiform initials are relatively rare and often are related with ray formation (Larson 1994).

Oblique anticlinal divisions, followed by intense intrusive growth of at least one of the derived sister initials (Bannan 1950; Evert 1961; Cumbie 1967; Srivastava 1973), occur in a domain pattern supposedly causing the rearrangement of cambial initials (Hejnowicz and Krawczyszyn 1969; Hejnowicz 1973; Hejnowicz and Romberger 1973). The number of these anticlinal divisions markedly exceeds the requirement for an adequate expansion of cambial circumference due to the increasing girth of the wood core (Bannan 1950; Evert 1961; Cumbie 1967; Srivastava 1973; Lim and Soh 1997a, b; Bossinger and Spokevicius 2018). The significance of this phenomenon, exclusive to the non-storeyed cambium, is still obscure.

The occurrence of excessive anticlinal divisions, followed by the supposed elimination of the initials produced in excess, was equated by Gahan (1988) and Mellerowicz et al. (2001) to the mechanism of somatic mutation elimination. Nonetheless, the question of why eliminations are so frequent in non-storeyed cambia and just seldom in storeyed cambia cannot be answered. Would it mean that non-storeyed cambium needs to eliminate somatic mutations more often, whereas storeyed cambium evidently does not?

Referring to the commonly reported high frequency of oblique anticlinal divisions in the non-storeyed cambium, which is far more than the actual requirement for the due expansion of the cambial circumference, Włoch et al. (2013) suggested that this excessive cell division could be the result of some specific pattern of mechanical strains occurring in the tissue. If the local maximal tensile stress in cell walls determines the orientation of the division plane (Louveaux et al. 2016), the occurrence of anticlinal division can possibly be a side effect of local mechanical stresses. The ability of the directed and synchronous intrusive growth in the storeyed cambium results in a rapid, coordinated change of fusiform initials’ orientation and inclination. That the coordinated response might cause a relaxation of shearing strains generated in the cambium (Włoch and Połap 1994; Kojs et al. 2004a, b; Jura et al. 2006). In the non-storeyed cambium, on the other hand, such a rapid rearrangement is not possible, and hence the magnitude of shearing strains exceeds this threshold, causing the initiation of excessive anticlinal divisions (Włoch et al. 2013).

In the storeyed cambium, an increase in circumference is usually considered to be an outcome of longitudinal anticlinal divisions (also known as radial longitudinal divisions) and the symplastic coordinated growth of the new sister initials with all initials (Miodek et al. 2021), whereas the rearrangement of initials is ascribed to the unidirectional apical intrusive growth of fusiform initials that occurs in numerous initial cells simultaneously but remains confined to cell ends, causing changes only to the position of the cell tips (Larson 1994; Włoch and Połap 1994; Kojs et al. 2004b; Evert 2006). The sister fusiform initials produced by the radial longitudinal divisions are almost equal in length, but their circumferential dimension (width) is halved, which increases subsequently by means of symplastic growth (Butterfield 1972). Thus, the radial longitudinal divisions in storeyed cambia contribute to the circumferential growth but do not affect the fusiform initials’ orientation and inclination—unlike the oblique anticlinal divisions of the non-storeyed cambia. The frequency of these anticlinal divisions is relatively high during the first few years of cambial activity, and decreases in the subsequent years (Bailey 1923; Butterfield 1972; Iqbal 1994; Kojs et al. 2004b), reflecting a decline in the relative increment of cambial circumference (Kojs et al. 2004b; Wilczek 2012).

The formation of a storeyed structure from a non-storeyed procambium is attributed normally to longitudinal anticlinal divisions (Cumbie 1984; Ajmal et al. 1986; Carlquist 1988; Soh 1990). It has long been held that the horizontal storeys of fusiform initials are homogeneous, and the intrusive growth of the initials is insignificant and unable to disturb a storeyed structure (Zagórska-Marek 1984). However, recent studies have revealed that although the directed intrusive growth does not disturb a storeyed structure, it does facilitate the vertical rearrangement of whole packets of initials, leading to the formation of heterogeneous storeys (Kojs et al. 2002; Wilczek 2012). This also provides an explanation for the rapid formation of regular storeys only during those few years of cambial activity when the frequency of anticlinal divisions is relatively low considering the large number of fusiform initials in a common storey. Tall multiseriate rays, if present, do not obstruct the spreading of regular storeys (Kojs et al. 2004b; Wilczek 2012; Miczajka et al. 2014). In several species with a storeyed structure of cambium, short oblique anticlinal divisions (often covering less than 60% of the cell length) are common in the first few years of cambial activity when storeyed structure is formed rapidly, and do not interfere with the process of storey formation (Kojs et al. 2004b; Wilczek 2012; Miczajka et al. 2014). The reports also indicate that such short anticlinal divisions become infrequent after regular storeys have been formed. All these facts make one wonder if the types of anticlinal division really determine the structure of cambium or, on the contrary, it is the structure of the cambium that actually defines the type of anticlinal divisions.

## Symplastic and Intrusive Cell Growths

### Symplastic Growth of Cambial Cells

Symplastic growth, typical for both the primary and secondary meristems, is a coordinated growth in which the various cells of a tissue grow in unison, keeping the mutual contacts with adjacent cells intact. Symplastic growth of cambial cell walls is anisotropic, being enormous in the radial direction, quite meager in the circumferential direction (causing a slight circumferential expansion), and nil in the axial direction (Karczewska et al. 2009; Miodek et al. 2021). The unequal extent of symplastic growth in the radial and circumferential directions is commonly accepted but often underestimated. A precise mathematical analysis of the cambium in tree trunk of 1 m circumference has revealed that, after a periclinal division, the daughter fusiform cells grow over 8000 times more in the radial direction than in the circumferential direction (Miodek et al. 2021). In fact, the circumferential increment of an individual initial due to the expansion of tangential walls is less than 0.002 μm, which is even less than the thickness of the cell wall (Karczewska et al. 2009; Miodek et al. 2021). In the example presented in Fig. 1a, after adding only one layer of cambial cells, hence increasing the cambial radius by 10 μm, the change of circumference per one initial would be equal to ΔCi = ΔC/N = 62.8 µm/5000 = 0.0126 µm, so less than the thickness of their radial walls. The relationship between the rates of growth in the radial and circumferential directions of an individual initial depends on the magnitude of the cambial radius or, more precisely, on the number of the initials participating in the increment of the cambial circumference.

In a study of active fusiform cambial initials, the intensely expanding areas of radial walls appeared to be completely devoid of cellulose (Roland 1978). Radial walls of fusiform initials consist mostly of hemicelluloses, as detected by Catesson and Roland (1981) and later confirmed by Catesson (1990). This agrees with the commonly accepted view of the maximal growth of cambial cells in the radial direction, which requires their radial walls to be especially suitable for rapid expansion through symplastic growth. Besides, the relative proportion of pectins and hemicelluloses also differs during the active and dormant phases of the cambium, as observed in *Populus tomentosa* (Chen et al. 2010). This may possibly be related to the difference in cell-wall extensibility. On the other hand, the nature of the tangential walls of fusiform cambial cells is rather cellulosic with a high content of methylated pectin (Catesson 1990; Mellerowicz et al. 2001). Such a structure seems to be insusceptible to stretching and expansion. In fact, the extent of symplastic growth of the tangential walls in the circumferential direction is quite insignificant in comparison to the extent of symplastic growth of the radial walls (Karczewska et al. 2009; Miodek et al.^2021^).

Catesson and Roland (1981) reported that the middle lamellae between the radial walls of fusiform cells were considerably thick, whereas those between the tangential walls of the initials and their closest derivatives were not even discernible. In a study of cytokinesis in cells of the root apical meristem, chemical composition of cell plate and middle lamella was found to be different (Matar and Catesson 1988). It is plausible that the middle lamella gains its mechanical strength over a period of time, and hence the attachment of recently divided cells (by periclinal divisions) is not strong enough to endure the tensile strain emerging rapidly after the sunset.

The relationship of microtubule arrangement with mechanical stress is well-documented (Hamant et al. 2008; Sampathkumar et al. 2014). In the first stage of tracheid differentiation, cellulose microfibrils are arranged longitudinally, whereas new microfibrils are later deposited in transverse orientation, a pattern similar to that observed in the arrangement of cortical microtubules (Abe et al. 1995). The longitudinal arrangement of microfibrils, predominant in the first stage of tracheid differentiation, seems to be related to cell expansion in the radial direction. The transverse arrangement of microfibrils possibly occurs when the radial expansion of tracheids has ceased, but a slight longitudinal expansion may continue (Abe et al. 1995; Funada 2008). The mechanism of regulation of the pattern of microtubule deposition and the reason of cell-wall growth taking place in one direction first and then in a different direction are yet to be worked out.

### Intrusive Growth and Its Location in Cambial Cells

Intrusive growth is meager in apical meristems and in differentiating primary tissues, but quite abundant in the elongate cells of the vascular cambium and their derivatives, i.e. elongating fibres, widening vessel-element mother cells, growing sieve-tube elements and several other cell types (Ghouse and Yunus 1975, Ghouse and Iqbal 1979; Iqbal and Ghouse 1983; Iqbal 1995; Lev-Yadun 2001; Gizińska et al. 2021). Here we focus only on the intrusive growth of the cambial initials. However, recent reports suggest that the same mechanical conditions are likely involved in the intrusive growth of cambial initials, vessel-element mother cells (Wilczek et al. 2011b; Gizińska et al. 2021) and fibres (Miodek et al. 2013; Wilczek et al. 2018).

**Fig. 3:**
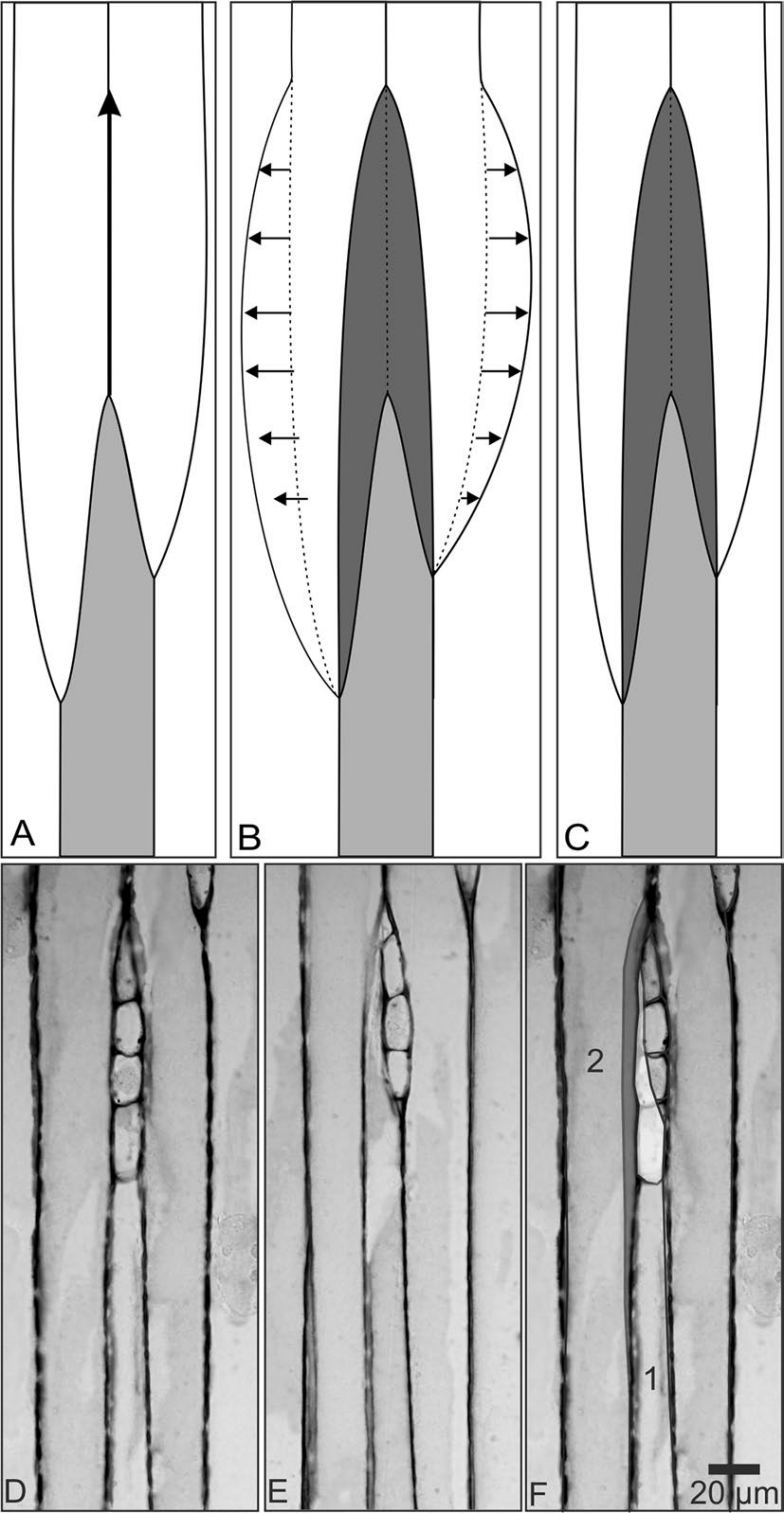
**A–F** Comparison of implications of the two hypotheses of apical intrusive growth of cambial initials on the arrangement of these initials as seen in tangential sections. **A**–**C** Schematics of three fusiform cambial initials: **A** Before the occurrence of intrusive growth in the lower initial (marked with grey) in the direction indicated by arrow; **B** After the occurrence of intrusive growth in the lower initial, assuming that it occurred between the radial walls of neighbouring initials and caused an increase in the cambial circumference. The intruding cell (marked with dark grey) pushed the contiguous cells sideways, as indicated by arrows, and caused an increase in the cambial circumference equal to the tangential surface of the intruding cell. The previous location of cell walls is indicated with a dotted line; **C** After the intrusive growth of the lower initial, assuming that it occurred along the tangential walls and caused no increase in the cambial circumference. The gain in the tangential surface of the growing initial (marked with dark grey) is equal to the loss (elimination) of the tangential surface of neighbouring initials. **D**–**F** Analysis of the arrangement of cambial cells of *Picea abies*, as seen in two tangential sections: **D** phloem mother cells, and **E** most likely cambial initials, sections obtained from positions 10 μm apart from each other; **F** both D and E seen together after superimposing. The superimposed view exhibits the area occupied by the intrusive growth of initials counterbalanced by an elimination of parts of the neighbouring initials marked: Dark grey—intrusive growth of initial 1, counterbalanced with the partial elimination of initial 2. Bright grey—intrusive growth of initial 1, counterbalanced with the partial elimination of two ray initials

The exact location of intrusion of the growing cambial initials has long been a subject of debate. It was initially assumed that intrusive growth contributes to the increment of the cambial circumference, hence the growing tip of the cell intrudes between the radial walls of the neighbouring initials (Larson 1994; Evert 2006). Comprehensive studies of rearrangement of cambial initials in tumours (Włoch 1976; Włoch et al. 2001) and in normal trunks of numerous trees (Włoch and Połap 1994; Włoch et al. 2002, 2009, 2013; Kojs et al. 2004a, b; Jura et al. 2006; Karczewska et al. 2009; Wilczek et al. 2011a) have questioned the long-held and commonly accepted explanation of the mechanism of intrusive growth. They suggest a new hypothesis, which identifies the occurrence of intrusive growth between the tangential walls of neighbouring initials and their closest derivative. This hypothesis (intrusion between tangential walls) provides a coherent explanation for the ‘elimination of initials’ also. It suggests that the intrusive growth of an initial is associated with an equal elimination (partial or total) of one (or more) neighbouring initials (Kojs et al. 2004a, b; Jura et al. 2006; Włoch et al. 2009, 2013; Wilczek et al. 2011a; Miodek et al. 2021) (Fig. 3). Such a growth is not likely to affect the circumference of the cambium, but it results in the rearrangement of cambial initials, plausibly to relax the strong mechanical shearing strains generated by the growing tissues (Kojs et al. 2004a, b; Jura et al. 2006; Wilczek et al. 2011a; Włoch et al. 2013).

Although the difference in the spatial location of intrusive growth seems to be of minor significance, it in fact leads to a dramatic change in our understanding of the functioning of the vascular cambium. Despite the repeated and thorough examinations of cambial structure in numerous studies, no instance of the actual increment of the cambial circumference could be detected during the intrusive growth of the cambial initials (Włoch et al. 2001, 2002, 2009, 2013; Kojs et al. 2004a, b; Jura et al. 2006; Karczewska et al. 2009; Wilczek et al. 2011a; Wilczek 2012; Miodek et al. 2021).

Earlier hypothesis assuming the occurrence of apical intrusive growth between the radial walls of adjacent initials was widely accepted and repeatedly mentioned in most of the literature dealing with the dynamics of cambial initials (Philipson et al. 1971; Iqbal and Ghouse 1990; Larson 1994). This was obviously in line with the concept that the intrusive growth of cambial initials was the main cause of circumferential expansion in the non-storeyed cambia (Cumbie 1963; Hejnowicz and Brański 1966; Iqbal 1994), due to the supposed intrusion of elongating initials between the radial walls of the neighbouring initials. Any such intrusion is possible only when the neighbouring initials lying ahead of the elongating cell tip are cleaved apart via dissolution of their middle lamella, and the intrusively growing initial fills the intercellular microspace thus produced; this would obviously increase the total circumference of the whole group of the initials (Fig. 3a, b).

The extent of increase to the cambial circumference due to the apical intrusive growth of a large number of anticlinally divided cells comes out to be much more abundant than is indeed required. This unusual situation was explained in the earlier literature by assuming that the excessive initials produced by anticlinal divisions were eliminated from the initial surface by their gradual shortening in length through successive unequal periclinal divisions and then differentiation of the reduced initials to become part of a derivative tissue (Zagórska-Marek 1984; Fahn 1990; Iqbal 1994; Larson 1994). The phenomenon of the so-called ‘elimination of initial’ (loss of initial), which is supposed to be of common occurrence in the initial layer of vascular cambium, also remains poorly explained (Larson 1994). Traditionally, the intrusive growth and the elimination of initials have been considered as two separate phenomena, the mechanisms of which could never be explained convincingly. The area of the eliminated initial was supposed to be filled gradually by the intrusively-growing adjacent initial (Zagórska-Marek 1984). However, it was never discussed what force pulls or pushes the adjacent initials towards the ‘space’ made available by the declining initial and helps them develop new connections with the adjacent cells and grow in unison.

However, if spaces previously occupied by the supposedly eliminated initials was later occupied by the intrusively grown tips of the neighbour initials, then the whole exercise of ‘elimination of initial’ becomes redundant because the supposed purpose of the phenomenon was to reduce the undue increase in the cambial circumference due to excessive intrusive growth.

The new concept of intrusive growth, on the other hand, explains that intrusive growth of an initial (along tangential walls) and so-called ‘elimination of initial’ via a simultaneous gradual disappearance of the adjacent initial occur inseparably and have no impact on the magnitude of the cambial circumference (Kojs et al. 2004a, b; Jura et al. 2006; Włoch et al. 2009, 2013; Wilczek et al. 2011a). As stated above, cambial tissue develops a tensile strain in the radial direction, caused by diurnal changes in tissue water balance. When this strain crosses some threshold level, it is likely that some areas of tangential walls are separated and fragments of at least some fusiform cells are detached from each other. When a fusiform initial grows into the space created that way, along the tangential walls of the neighbouring one they may be envisaged to be in a temporary competition for the same area of the initial surface. In this competition, one initial loses its initial status and is moved away from the initial surface by the symplastically growing tissue. If a whole initial moves away from the initial surface, it would appear to have undergone a total elimination from the initial surface, and the area which was previously occupied by this initial is now occupied by the intrusively growing initial (Włoch et al. 2009, 2013). This new arrangement of initials is established further by the prospective periclinal divisions, which are unequal, resulting in a smaller (after an elimination) and a larger (due to intrusive growth) initial (Fig. 3a, c). Two tangential sections of phloem mother cells and the most probable cambial initials were superimposed (Fig. 3d–f). Phloem (or xylem) mother cells reflect the arrangement of cambial initials existing when that layer of cells was deposited, hence may be considered as a record of the past. Initials present the most actual arrangement of the cambial cells (Kojs et al. 2004b; Jura et al. 2006; Włoch et al. 2013; Wilczek et al. 2011a). One fusiform initial has grown intrusively, but the location of radial walls of neighboring cells remains identical except for the area of intrusive growth. The intrusively growing initial didn't push any other initial away, but instead occupies the area previously occupied by the neighbouring fusiform initial, partially eliminating it from the initial surface. It has also eliminated one ray initial and partially eliminated three others. Similar examples has been presented in numerous studies (Kojs et al. 2004b; Jura et al. 2006; Włoch et al. 2013; Wilczek et al. 2011a).

**Fig. 4:**
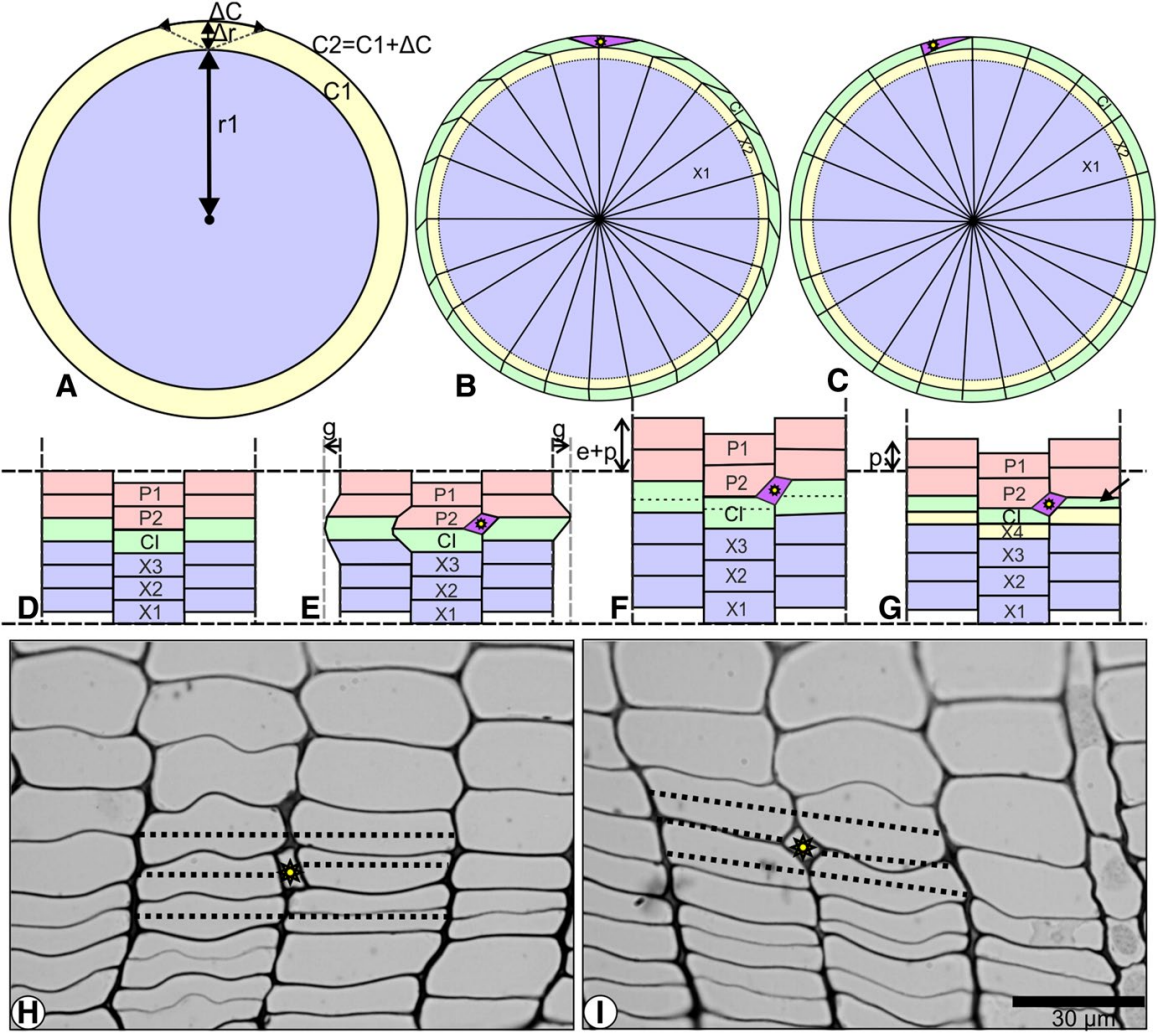
**A**–**I**. Implications of two hypotheses regarding the intrusive growth of fusiform cambial initials as seen in transverse sections: **A** Relationship between the radial and circumferential increments of the cambium. Cambium with a given radius (r1) and circumference (C1) before the occurrence of intrusive growth. Change in the cambial circumference (ΔC) has to be associated with the proportional (ΔC = 2ΠΔr) change in its radius (Δr). The proportions between Δr and ΔC are modified for better visibility. C1- inner circle, C2—outer circle. **B** Based on the old hypothesis that intrusive growth contributes to the increment in the cambial circumference; the arrangement of radial files was disrupted by the intrusion of the growing initial. **C** Based on the new hypothesis that intrusive growth causes no increment in the cambial circumference; the arrangement of radial files is regular despite the occurrence of intrusive growth of the initial, except for the area covered by intrusive growth. P1-P2—layers of deposited phloem mother cells; X1-X3—layers of deposited xylem mother cells; Radial lines—radial files; CI—thickness of initial layer; asterisk—intrusively growing initial. **D**–**G** Diagrammatic presentation of the impact of intrusive growth on the arrangement of three adjacent radial files of cambial cells: **D** Arrangement of cambial cells before the occurrence of intrusive growth; **E** Based on the hypothesis that intrusive growth causes an increment in the cambial circumference. The same three radial files of cambial cells (as on Fig. D) after intrusion of one fusiform initial between the radial walls of neighbouring initials. Grey dashed vertical lines—increased tangential dimension of radial files (look at g above the arrows). **F** and **G** Based on the hypothesis that intrusive growth causes no increase in the cambial circumference; **F** The same three radial files of cambial cells (as in Fig. D) as seen after the intrusive growth of one fusiform initial. The cambial zone is elastically (e) and plastically (p) tensed in the radial direction (during the night). Locations of the forthcoming periclinal divisions are marked with dashed horizontal lines. **G**) The same three radial files (as in Fig. f) after periclinal divisions, resulting in the deposition of a new layer of xylem mother cells (X4). The periclinal division marked with an oblique arrow is unequal). **H**, **I** Two examples of intrusive growth observed in transverse sections of a *Picea abies* cambium display no disruption of radial files. The intrusion of the initial (marked with asterisks) does not add to the circumferential dimension of the group of initials, but causes a corresponding reduction in the dimensions of neighbouring initials (marked with dashed lines). Asterisk—intrusively growing initial; Dashed horizontal lines—tangential dimension of radial files

The arrangement of cell walls in the radial file should also be analyzed to determine whether the intrusive growth of cambial initials contributes to the increase in the cambial circumference. As the cambial tissue normally occurs in a more or less cylindrical form, any increase in the circumference of this cylinder has to be linked to a corresponding increment of its radius, following the rules of geometry (Fig. 4).

In a single radial row of cambial cells, which is a very small segment of the cambial circumference, it is not possible to observe the curvature of the periclinal walls (Figs. 1, 4) and usually a term 'tangential plane' is used to describe a position of dividing wall in periclinal division. Plant anatomists use this term conventionally while measuring the width of cambial cells on anatomical preparations normally referred to as the tangential, radial and transverse sections. However, from mathematical point of view, the term ‘tangential’ may perhaps be replaced with ‘circumferential’ while referring to this dimension of cells in a tissue of cylindrical form such as the vascular cambium.

If, according to the previous concept, intrusion of an elongating fusiform initial occurs between the radial walls of contiguous initials, then the intrusive growth of every single cell would make some addition to the cambial circumference. Theoretically we can assume two possibilities: such a sudden increment of circumference could be local, without any impact on neighbouring radial files, or it would have an impact on these files. Any localized increment in a sector of cambial circumference, without a corresponding radial increment of the whole tissue, would likely form a sort of bulge on the initial layer, but nothing like this has ever been observed in microscopic studies. Moreover, in a cylindrical tissue, any increase in the cambial circumference, resulting from the intrusive growth of even one initial, has to be accompanied by radial increment of the tissue. Suppose the intrusive growth of only one initial (ΔC) adds, for instance, 20 μm to the cambial circumference, then the radius of the cambial cylinder should correspondingly increase by 3.18 μm (i.e. Δr = ΔC/2π = 20 μm/2 × 3.14 = 3.18 μm). Massive occurrence of intrusive growth should mean many times more growth in radial direction (in proportion to the size of the circumference). So such massive intrusive growth would have to be accompanied by an adequate simultaneous increase in the cambial radius.

Besides, if the growing initial intrudes along the radial walls of adjacent initials, the circumferential dimension of all cambial cells located lateral to the intrusively grown initials should remain constant (unaffected). However, this is never the case, and the tangential width of the initials lateral to the intrusively grown one is invariably and proportionately reduced. Moreover, in the case of an initial intruding along the radial surface of adjacent initials and adding to the cambial circumference, not only these adjacent cells would be cleaved apart by this intrusion, but the next neighbours would also be pushed laterally, causing a dislocation of initials all along the cambial circumference. That would be the case especially for non-storeyed cambia, where intrusive growth was commonly mentioned as the main way of circumferential increment. This dislocation is bound to disturb the alignment of cambial initials with the radial files of their derivatives (Fig. 4b). It would not be logical to presume that all the other initials might decrease their tangential dimensions in order to maintain the proper alignment of radial files; such an assumption would also nullify the presumed impact of intrusive growth on the cambial circumference. However, according to the new hypothesis, the intrusive growth of an elongating initial occurs between the tangential walls of the neighbouring initial and its immediate derivative and is linked with an equal elimination of the neighbouring initial from the layer of initials (or the initial surface). Therefore, the arrangement of radial files remains intact, except in the area of intrusive growth (Fig. 4c).

Figure 4d and e exhibit the impact of intrusive growth estimated on the basis of old hypothesis, supposing that intrusive growth is the mechanism for increase in the cambial circumference. This hypothesis does not explain the relationship between the symplastic growth of the whole cambial tissue and the intrusive growth of a particular fusiform initial. As we can see in Fig. 4b, the cambial tissue has increased its circumference and radius, which obviously means that the volume of the xylem cylinder has increased. An increment in cambial radius is commonly accepted to be the result of symplastic growth and periclinal divisions of cambial initials and their derivatives. This too is well accepted that, in the case of storeyed cambia, anticlinal division and symplastic growth of initials are responsible for the circumferential increment of the cambium. However, in non-storeyed cambia, the same outcome is surprisingly attributed to intrusive growth rather than to symplastic growth. Nonetheless, it has never been explained how the intrusive growth of even one initial would coordinate with the normal symplastic radial expansion of the cambial tissue. Further, the supporters of the concept of ‘elimination of initials’, should not ignore the fact that every local/sectorial change, taking place anywhere in the cambial cylinder, would have an impact on the overall circumference of the cambium. And this would also cause a proportional change in the radius of the wood and the cambial zone.

According to the new hypothesis, intrusive growth is inseparably linked to the symplastic growth of the tissue, because both these features are the outcome of one fundamental process, i.e. the diurnal variation of water balance, which causes a change in phloem radius. This hypothesis asserts that the cambial zone is tensed in the radial direction when the secondary phloem swells, typically during the night. The tension may likely be a combination of elastic and plastic strains (Fig. 4f) (Kojs and Rusin 2011; Miodek et al. 2021). This radial tension, which was not taken into consideration earlier, explains the assumed formation of space available for intrusive growth between the tangential walls. If the tangential walls of any initial and its immediate derivative are separated (for instance because of shearing strains), the tension would move these cells away from each other. In the space thus produced, one of the neighbouring initials may grow intrusively. The following day, when the phloem decreases its turgor pressure, the radial tension prevailing in the cambial zone during the night, will decrease. The elastic strain is then withdrawn and the plastic strain remains intact in the form of a radial symplastic increment of the cambial tissue (Fig. 4g).

As the intrusive growth of one initial is counterbalanced by the corresponding elimination of a neighbouring initial(s) (Fig. 4h, i), no disruption of radial files is visible in the transverse sections of the cambial zone. This corroborates the new hypothesis, which proposes intrusion of the growing initial along the tangential walls of the neighbouring cells, causing no increment in the cambial circumference.

**Fig. 5:**
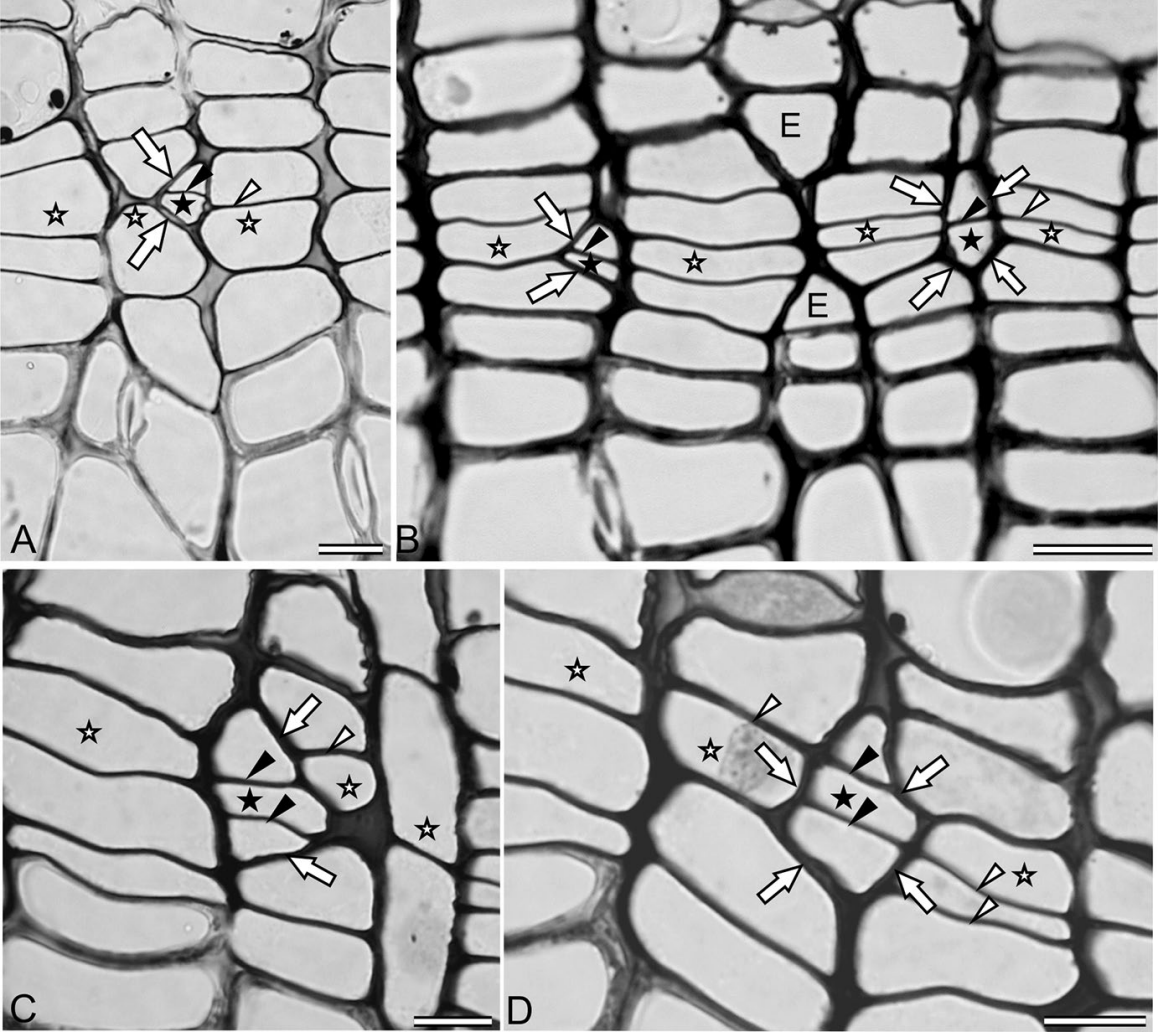
Transverse sections of the cambium of *Pinus sylvestris,* depicting the initials, the tangential walls of which have become slanted due to the intercellular intrusion of neighbouring initials. White arrows indicate slanted walls during their transformation from tangential walls to anticlinal ones. Black arrowheads point to tangential walls formed due to unequal periclinal divisions of cells of a new radial file, whereas white arrowheads show tangential walls formed due to unequal periclinal divisions of cells of the eliminating radial file. Asterisks indicate the most probable initial cell; Black asterisks—intrusively growing initial; E—elimination of radial file due to the lateral intrusive growth of two neighbouring initials. Scale bars—10 μm. (Similar examples were presented by Jura el al. 2006; Karczewska et al. 2009)

In transverse sections of the active cambial tissue, with its cells dividing periclinally and growing symplastically as well as intrusively, one often comes across some slanted walls of the initials that have undergone intrusive growth or elimination, indicating a gradual transformation of their tangential walls into radial walls (Figs. 1, 4h, i, 5). Such a transformation (of tangential walls into radial ones) has been observed in areas with vigorous intrusive growth in the cambia of both the gymnospermous and dicotyledonous species (Jura et al. 2006; Włoch et al. 2009; Wilczek et al. 2011a). The number of layers of cambial cells in the space surrounded by the slanted walls of the initials reflects the relative duration for which the intrusive growth has been in progress in the given area of cambial tissue. If the intrusive growth has occurred immediately before the sample collection, the slanted walls should be confined within one layer of cells (Fig. 4h, i). However, the number of the cell layers in contact with slanted walls increases with time due to the consistent symplastic growth of cells and the concurrent incidence of periclinal division (Figs. 4, 5). Newly produced tangential walls connect the slanted walls, just as they connect the radial ones at the other end. The space surrounded by the slanted walls (i.e. the transactional area of the intruding cell) shows unequal periclinal divisions, wherein dimensions of the cell-plate area change according to the growing dimensions of the cell concerned. In the new radial file formed by the intrusively grown initial, cells and cell plates are wider (in the circumferential direction), whereas in the radial file of the eliminated initial, they are narrower in the circumferential direction (Fig. 5). If the elongating initial has intruded between a neighbouring initial and its immediate derivative, the slanted walls appear on one side of the new radial file and form a shape similar to a triangle (Fig. 5a, c). If, on the contrary, the initial has intruded between two neighbouring initials and their immediate derivatives, the slanted walls form a rhomboidal shape (Fig. 5d). In some cases, intrusive growth occurs not around the tip of the initials, but on their lateral walls. Figure 5b exhibits this condition, where one radial file is eliminated because of the lateral growth of both of the neighbouring initials. Such eliminations of radial files have often been described earlier without any reference to intrusive growth.

It is curious to assume that the tangential walls of adjacent cambial cells are temporarily separated from each other due to intrusion of some elongating cells between them, but it finds support from certain observations, such as the occurrence of unequal distribution of plasmodesmata on the radial and the tangential walls. Plasmodesmata hardly occur on the tangential cell walls of fusiform initials, but are abundant on their radial walls (Catesson 1990; Ehlers and van Bel 2010). This is exactly what would be expected on assuming (a) a frequent separation of tangential walls of cells in a radial file, and (b) a firmness of association between the radial walls of cells of the contiguous radial files. This uneven distribution and frequency of plasmodesmata makes one think that either the frequent separation of the newly produced tangential walls disrupts the newly formed plasmodesmata, or the plasmodesmata are rarely formed on the tangential walls, possibly as an adaptation to the frequent separation of these walls.

One of the characteristic features of meristematic cells is the absence of large vacuoles. However, cambial initials normally possess one big vacuole per cell during the active phase and a few smaller ones during the dormant phase of the cambium (Iqbal and Ghouse 1985). The vacuolar compartment seems to have a role in the process of xylogenesis, but its significance in the cambial initials is still unclear (Arend and Fromm 2003). The intrusive growth of cambial initials may be intense, involving a rapid enlargement of cell volume and wall surface. In this process, further enlargement of an already extended vacuolar system related to the active uptake of osmotically active solutes seems to be helpful (Arend and Fromm 2003). It was proposed that intrusive growth occurs on the edge of the cell with the smallest turgor pressure and hence the intrusively growing cambial initial cannot actually cleave the adjacent cells apart, and can only fill the already existing microspace (Hejnowicz 1980). Intrusive growth should therefore be a passive phenomenon, unable to separate the walls of two neighbouring cells, and hence may occur only in areas with previously formed microspaces between the walls of adjacent cells (Kwiatkowska and Nakielski 2011). The role of vacuoles in the regulation of turgor pressure, which could possibly allow for intense intrusive growth, may be an interesting subject for future research.

### Role of Intrusive Growth in Rearrangement of Cambial Initials

The circumferential expansion in the storeyed and non-storeyed cambia is believed to occur by different mechanisms, i.e. through longitudinal anticlinal divisions coupled with the symplastic growth of the resultant sister initials in storeyed cambium, while through oblique anticlinal divisions followed by intrusive growth of the sister initials in the case of non-storeyed cambium (Fahn 1990; Larson 1994; Evert 2006). Such a unique situation would require some curious arrangement for a simultaneous regulation or a periodical switching over between the two different mechanisms, which has never been observed. As the storeyed structure of cambium develops from the non-storeyed procambium during the plant ontogeny (Soh 1990; Larson 1994; Evert 2006), one may genuinely expect coexistence of both the storeyed and non-storeyed cambial structures in a given specimen. Furthermore, in the so-called mosaic cambium, both types of the cambial structure do occur together (Krawczyszyn 1977). The increase of cambial circumference in such cases should, therefore, involve two different mechanisms, one of which is dependent on symplastic growth and longitudinal divisions, while the other is dependent on the oblique anticlinal divisions and intrusive growth of the initials.

Recent reports have shown a non-participation of intrusive growth in the circumferential increment (Kojs et al. 2004a, b; Jura et al. 2006; Włoch et al. 2009, 2013; Wilczek et al. 2011a). In such a situation, symplastic growth that follows the anticlinal division is the only alternative that may provide a common and uniform mechanism for the increase in cambial circumference, both in the storeyed and non-storeyed cambia.

Cell rearrangement in non-storeyed cambia was ascribed to oblique anticlinal divisions followed by intrusive growth, whereas typically in storeyed cambia oblique anticlinal divisions don't occur and intrusive growth was considered insignificant. If oblique anticlinal divisions, followed by intrusive growth of the cells produced, constitute the main mechanism for the rearrangement of cambial initials, the intensity of cell rearrangement should be markedly less in storeyed cambia, which hardly exhibit the occurrence of oblique anticlinal divisions (Larson 1994; Evert 2006). Nonetheless, the most rapid rearrangement is seen in the storeyed cambia, particularly in old trunks where oblique anticlinal divisions are practically absent (Krawczyszyn and Romberger 1979; Kojs et al. 2002, 2003, 2004b; Włoch et al. 2013). This also indicates that significance of oblique anticlinal divisions as the possible mechanism for rearrangement of cambial initials has been highly overestimated. In consequence of the new hypothesis, intrusive growth is supposed to be the mechanism responsible for rearrangement of cambial initials in both the storeyed and non-storeyed types of cambium (Włoch et al. 2001, 2002, 2013; Kojs et al. 2004a, b; Wilczek, 2012; Wilczek et al. 2011a).

An oblique anticlinal division of fusiform initials results in two shorter initials. Interestingly, numerous such divisions are followed by the elongation of one of the sister fusiform initials produced, often with a total or partial elimination of the other sister initial. The intrusively growing sister initial increases its axial dimensions, but the other sister initial appears to undergo a simultaneous thinning and/or shortening, and may ultimately disappear from the layer of initials, as described in numerous reports (Bannan 1950; Evert 1961; Cumbie 1967; Srivastava 1973; Lim and Soh 1997a, b; Bossinger and Spokevicius 2018). This apparent thinning or shortening of initials is in fact a case of overlapping of the adjacent initial(s) by the intrusively growing initial along the tangential surface of the contiguous cells. This supports the view that the apical intrusive growth of cambial initials takes place along the tangential walls rather than the radial walls of neighbouring cells lying ahead of the elongating cell tip (Jura et al. 2006; Karczewska et al. 2009). In this situation, despite the fact that the growing initial gains in its tangential dimension (cell width), no increase accrues to the cambial circumference.

In the non-storeyed cambia, especially in gymnosperms, intrusive growth covers a larger area of the initial, whereas in storeyed cambia it is confined to a small area around the cell end, merely causing a change in the cell-tip location, although the frequency of such events is very high (Krawczyszyn and Romberger 1979). This could be why the significance of apical intrusive growth for cellular rearrangement in storeyed cambia has been underestimated. Such a rearrangement involves a synchronic intrusive growth at the tip of large groups of cambial initials arranged in storeys, together with the concurrent fusion and splitting of rays (Krawczyszyn and Romberger 1979; Włoch and Szendera 1989; Kojs et al. 2004a). The small but synchronic changes of inclination in large groups of initials arranged in horizontal storeys have an enormous impact on the inclination of the cambial derivatives, which imitate the structural pattern of the cambium. The cambial structure in which the initials change their inclination rapidly and synchronically is called a ‘functional storeyed structure’ (Kojs et al. 2002; Włoch et al. 2013).

Recent observations and their interpretations suggest that the significance of oblique anticlinal divisions requires a critical reappraisal (Włoch et al. 2013). It was previously assumed that oblique anticlinal divisions followed by intrusive growth constitute the main mechanism operative behind the rearrangement of cambial initials. The basis of this assumption was the belief that the intrusive growth of elongating cells takes place between the radial walls. Given this, a lack of new oblique radial walls would make the intercellular intrusion of a growing cell tip, and hence the consequent rearrangement of cells, impossible. However, if the newly formed initials elongate by intrusive growth along the tangential surface of adjacent cell(s), and the radial walls have no role in the process, the frequency of anticlinal divisions becomes irrelevant in this regard. Therefore, the supposed relationship between oblique anticlinal divisions and cambial cell rearrangement needs to be re-evaluated.

## Conclusions

Recent studies on the radial growth of arborescent plants have brought out the following facts related to the cambial cell dynamics:

1. The frequent periclinal divisions of cambial cells contribute adequately to the radial expansion of the symplastically growing cambium tissue by adding new layers of derivatives. The radial dimension of cambial cells is maintained by symplastic growth of the radial walls of daughter cells after each periclinal division.
2. The symplastic growth of cambial cells after periclinal division occurs mainly on radial walls (in the radial direction), and meagerly on tangential walls (in the circumferential direction), corresponding to the ratio between the increase in the radius of the wood core and the resultant increase in the circumference of the cylindrical tissue of cambium. This is the only mechanism of radial and circumferential growth because of the activity of the vascular cambium.
3. The much less frequent anticlinal divisions of cambial initials contribute to the required expansion of the cambial circumference and ensure the maintenance of normal tangential dimensions of cambial initials throughout synchronic symplastic growth of all initial cells. The excess of oblique anticlinal divisions, observed normally in non-storeyed cambia, may plausibly be the result of some specific strains in the cambial tissue. They have no direct impact on the dimensions of the cambial cylinder or the arrangement of the cambial initials because the excess initials produced by these divisions are eliminated from the layer of initials due to the apical intrusive growth of their adjacent initials along the tangential wall surface.
4. The intrusive growth of cambial initials, which has long been regarded as the main mechanism of increase in the cambial circumference of non-storeyed cambia, has in fact no role in that process. It occurs (in the axial and tangential directions) between the tangential walls of the adjacent initial and its immediate derivative, and is always counterbalanced with an equal amount of eliminations of the neighbouring initials, irrespective of the frequency of anticlinal divisions. It does, however, result in the rearrangement of cambial initials. Such locations of intrusive growth in the cambium indicate a complete or, as a result of unequal periclinal divisions, a partial loss of the initial status. Thus, there is nothing like ‘elimination’ or ‘loss’ of the initial cells in actuality; it is only a case of pushing the cell from the initial surface of the cambium to the derivative tissue.
5. Mechanical strains in the vascular cambium keep changing in diurnal cycles, due to alteration in water balance and hence in turgor pressure of cells in the derivative tissues.
